# Nursing theories developed to meet children’s needs: a scoping review

**DOI:** 10.1590/1980-220X-REEUSP-2022-0151en

**Published:** 2022-09-09

**Authors:** Ana Márcia Nóbrega Dantas, Renata Clemente dos Santos-Rodrigues, José Nildo de Barros Silva, Maria Naiane Rolim Nascimento, Marcos Antônio Gomes Brandão, Maria Miriam Lima da Nóbrega

**Affiliations:** 1 Universidade Federal da Paraíba, Centro de Ciências da Saúde, Programa de Pós-Graduação em Enfermagem, João Pessoa, PB, Brazil.; 2 Universidade Federal do Ceará, Programa de Pós-Graduação em Enfermagem, Fortaleza, CE, Brazil.; 3 Universidade Federal do Rio de Janeiro, Departamento de Enfermagem Fundamental, Rio de Janeiro, RJ, Brazil.

**Keywords:** Nursing Theory, Models, Theoretical, Grounded Theory, Child, Child Care, Nursing, Teoría de Enfermería, Modelos Teóricos, Teoría Fundamentada, Niño, Cuidado del Niño, Enfermería, Teoria de Enfermagem, Modelos Teóricos, Teoria Fundamentada, Criança, Cuidado da Criança, Enfermagem

## Abstract

**Objective:**

To map the nursing theories developed to meet children’s needs.

**Method:**

The JBI methodology and the PRISMA guided this scoping review. The search took place between October and November 2021, based on the PCC mnemonic: P (Population) – a child aged 0 to 9 years; C (Concept) – nursing theories; C (Context) – nursing.

**Results:**

We identified 2,242 publications and selected 21 manuscripts consisting of nursing theories to meet children’s feeding needs, child pain, a child with asthma, diabetes, obesity, epilepsy, Congenital Zika Syndrome, autism spectrum disorder; theory for care in Intensive Care Units; health promotion to premature babies; procedures in Intensive Care Units; theory for nursing diagnosis ineffective breathing pattern in children with congenital heart disease; sleep associated with child development; parent-child interaction; nurse-child relationship; and child’s consultation.

**Conclusion:**

The nursing theories mapped have the potential to outline the course of nursing care to children’s needs highlighted in the studies that made up the sample.

## RESUMO

**Objetivo:**

Mapear as teorias de enfermagem desenvolvidas para atender às necessidades infantis.

**Método:**

Estudo do tipo *scoping review*, orientado pela metodologia JBI e pelo roteiro do PRISMA para revisões de escopo. A busca ocorreu entre outubro e novembro de 2021, norteada pelo mnemônico PCC: P (*Population*) – criança com idade de 0 a 9 anos; C (*Concept*) – teorias de enfermagem; C (*Context*) – enfermagem.

**Resultados:**

Foram identificadas 2.242 publicações e selecionados 21 manuscritos, constituidos por: teorias de enfermagem para atender às necessidades de alimentação da criança, de dor infantil, criança com asma, diabetes, obesidade, epilepsia, síndrome congênita pelo vírus Zika, Transtorno do Espectro Autista; teoria para o cuidado na Unidade de Terapia Intensiva; promoção da saúde a bebês prematuros; procedimentos na Unidade Terapia Intensiva; teoria para diagnóstico de enfermagem padrão respiratório ineficaz em crianças com cardiopatias congênitas; sono associado ao desenvolvimento infantil; interação pai-filho; relação enfermeiro-criança; e consulta infantil.

**Conclusão:**

As teorias de enfermagem mapeadas têm o potencial de delinear o percurso do cuidado de enfermagem às necessidades da criança destacadas nos estudos que compuseram a amostra.

## RESUMEN

**Objetivo:**

Mapear las teorías de enfermería desarrolladas para atender las necesidades de los niños.

**Método:**

Un estudio de revisión de alcance, guiado por la metodología JBI y la hoja de ruta PRISMA para revisiones de alcance. La búsqueda se realizó entre octubre y noviembre de 2021, guiada por el mnemónico PCC: P (Population) – niño de 0 a 9 años; C (Concepto – teorías de enfermería; C (Context) – enfermería.

**Resultados:**

Se identificaron 2242 publicaciones y se seleccionaron 21 manuscritos, consistentes en: teorías de enfermería para atender las necesidades de alimentación del niño, dolor infantil, niño con asma, diabetes, obesidad, epilepsia, síndrome congénito del virus Zika, desorden del espectro autista; teoría para el cuidado en la Unidad de Cuidados Intensivos; promoción de la salud de los bebés prematuros; procedimientos en la Unidad de Cuidados Intensivos; teoría para el diagnóstico de enfermería patrón respiratorio ineficaz en niños con cardiopatías congénitas; sueño asociado con el desarrollo infantil; interacción padre-hijo; relación enfermera-hijo; y consulta infantil.

**Conclusión:**

Las teorías de enfermería mapeadas tienen el potencial de delinear el curso de la atención de enfermería a las necesidades del niño destacadas en los estudios que componen la muestra.

## INTRODUCTION

Changes and evolutions in nursing knowledge influenced the perception of the relevance of nursing theories. Initially, considered as an element that would give greater scientificity to the subject, over time, were contested inside and outside nursing as being too abstract and without a specific context, especially grand theories, including notes of difficulty of application in nursing practice^(1)^. Nursing has improved through methods and research^(2)^. It is claimed that some success seems to have been achieved with the proposal of narrower scope theories.

Nursing theories are oriented to specific phenomena in the sphere of nursing. Thus, they produce key ideas that express the essence of nursing practice, providing a comprehensive and informed understanding^(3)^. Moreover, they bring a relevant contribution to nursing as a science by shaping their body of knowledge with theoretical and practical content to guide nurses in patient care, in addition to providing a basis for research in the form of a reference or advancing through validation by empirical testing. They are more specific than the conceptual model and aim to explain, describe, predict or prescribe the phenomenon as a whole from a nursing perspective^(4)^.

Research on nursing theory development continues to be a topic of interest in the subject, as studies have shown. In Jordan, 11% of nursing theories were used in research^(5)^. In Qatar, nursing theories were frameworks for 14% of research^(6)^. In Portugal, just over 7% of theses and dissertations used nursing theories as a framework^(7)^. In Mexico, just over 13% of articles used nursing theories^(8)^.

An integrative review in English used nursing theories as frameworks for experimental studies, demonstrating the use of theories to guide the research. More than 68% applied the Self-Care Deficit Theory and the Adaptation Model^(9)^. In Brazil, a study showed that just over 9% of theses and dissertations included in the research used nursing theories in their development, of which the theories that predominated were: Transcultural Theory (12.7%); Theory of Basic Human Needs (11.1%); and Theory of Praxis Intervention in Nursing in Collective Health (TIPESC) (11.1%)^(10)^.

Although these and other studies indicate the use of nursing theories, only one study, produced in Brazil, indicated human group data. In this research, the authors found that child health care was the theme for 31.4% of the studies that used theories. However, it is not clear whether such studies used theories cut to this human group^(10)^. Based on this insufficient characterization of the use of theories aimed at child care, the present research is justified.

The objective of this study was to map the nursing theories developed to meet children’s needs.

## METHOD

### Study Design

A scoping review study with a research protocol registered in the Open Science Framework (https://osf.io/29cts/), identification DOI: 0.17605/OSF.IO/29CTS: DOI, guided and structured by the JBI^(11)^ methodology and the PRISMA roadmap for scoping reviews^(12)^. Five steps were taken: research question identification, relevant study identification, study selection, data analysis, data synthesis, and presentation^(12)^.

### Research Question Identification

The guiding question, the objective of the study, and the descriptors were based on the PCC mnemonic combination: P (Population) – a child aged 0 to 9 years; C (Concept) – nursing theories; C (Context) – nursing. The guiding question was: how are nursing theories built to meet children’s needs characterized?

### Selection Criteria

We included studies such as books, original articles, theses, and dissertations, encompassing content on nursing theories developed to meet children’s needs and presented in nursing scientific evidence. No temporal or idiomatic cut was used, as recommended by the JBI methodology. We exclude duplicate studies that are not fully accessible in open or restricted databases.

### Search Strategies

The search took place between October and November 2021 by two independent reviewers. In the first search, the crossing (Child AND “Nursing Theory” AND Nursing) was applied, corresponding to PCC in the PubMed and (*Criança* AND “*Teoria de enfermagem*” AND *Enfermagem*) SciELO databases, to identify the descriptors and terms corresponding to the acronyms.

Through this first investigation, it was possible to compose the specific search strategy, relating the identified descriptors and Boolean operators AND and OR: Chid (“Infant” OR “Children” OR “Young children” OR “Pediatrics” OR “Child Health” OR “Newborn” OR “Neonatology” OR “Preschool” OR “Infants” OR “Minors” OR “Child Care”) AND Nursing Theory (“Middle-range theory” OR “Theoretical model” OR “Models, Nursing” OR “Theory Construction” OR “Theory Building” OR “Theory development” OR “Models, Theoretical” OR “Specific situation theory” OR “Models, nursing” OR “Grounded Theory”) AND Nursing (“Pediatric nursing” OR “Nurse’s role” OR “Nursing care” OR “Children’s nursing” OR “Nursing Staff” OR “Nurse-Patient Relations” OR “Neonatal Nursing”).

For the strategy mentioned above, the following databases were used: Cumulative Index to Nursing and Allied Health Literature (CINAHL), Web of Science (WHO), MEDLINE^®^ (PubMed^®^), Scopus (Elsevier), Scientific Electronic Library Online (SciELO), and Latin American and Caribbean Literature in Health Sciences (LILACS). Gray literature evidence was investigated in the Coordination for the Improvement of Higher Education Personnel (CAPES – *Coordenação de Aperfeiçoamento de Pessoal de Nível Superior*) Theses and Dissertations Catalog, Google Scholar, research on websites of official bodies, manuals of international and national institutions, guidelines and books. Finally, we made a reference list of selected documents to identify new research related to the study topic.

### Study Selection

They were grouped and exported to Rayyan QCRI to carry out article selection and reduce selection bias. Studies in more than one database were considered only once by order of identification in the first observed database. The themes that received divergent assessments were resolved by a third author, culminating in the sample considered for reading in full.

### Data Gathering

Data were extracted using a specific form created by the authors of this article. The document contained the study characterization (authorship, title, year, study design, country, journal, database) and theory characterization (level of abstraction, a method used to build the theory, strategy, and purpose for developing the idea, approach, predominant reasoning, implications for nursing, in addition to concepts, statements, and theorization). This information was entered into a Microsoft Office Excel^®^ 2019 spreadsheet, supporting the synthesis and description of the results with the scope of the guiding question.

### Data Synthesis

We entered the collected data in the charts presented in this article’s results to facilitate the understanding and visualization of theories. The discussion aimed to assess the scope of each identified theory. The results are presented in tables in descriptive format, followed by an explanatory summary.

### Ethical Aspects

This review research did not require approval by a Research Ethics Committee for its development.

## RESULTS

**Figure 1.**
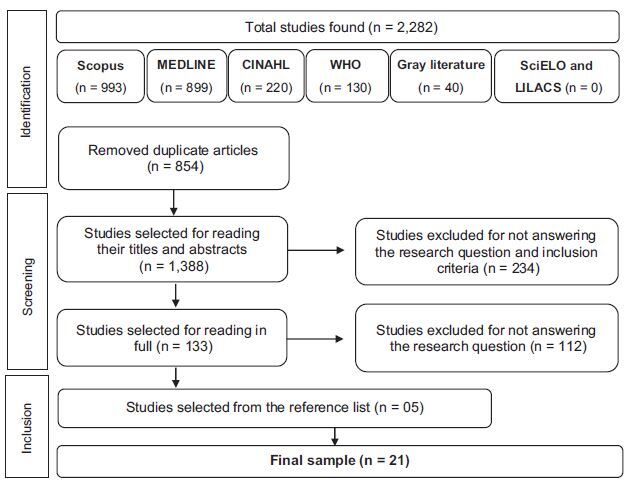
Flowchart adapted from PRISMA for scoping reviews for study selection and inclusion process, used to select the results – João Person, PB, Brazil, 2022.

We identified 2,282 articles, of which 1,388 were selected after the exclusion of duplicate studies, for reading their titles and abstracts. Subsequently, 133 articles were read in full, as they had content addressing nursing theories developed for child care, culminating in a final sample of 21 articles (Figure 1).

**Chart 1.**
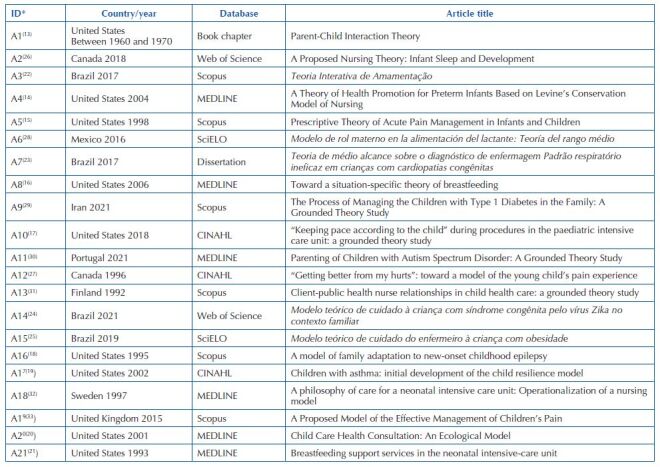
Characterization of articles that make up the study sample according to country, year, databases, title – João Pessoa, PB, Brazil, 2022, n = 21.

Chart 1 shows the selected studies according to country, year, databases, and title. Of the 21 publications, nine studies (42%) were conducted in the United States^(13–21)^, four (19%) in Brazil^(22,23,24,25)^, two (9%) in Canada^(26,27)^. Countries such as Mexico^(28)^, Iran^(29)^, Portugal^(30)^, Finland^(31)^, Sweden^(32)^, and the United Kingdom^(33)^ presented only one (5%) study each. About the databases in which they were identified, six (29%) were in Scopus^(15,18,22,29,31,33)^ and MEDLINE^(14,16,20,30,32)^, respectively. The CINAHL^(17,19,27)^ database contained three (14%) studies. Web of Science^(24,26)^ and SciELO^(25,28)^ presented two (10%), respectively, also observing a study in book chapter format and a dissertation. The articles whose database was SciELO were identified while reading the article references.

ID: identification.

**Chart 2.**
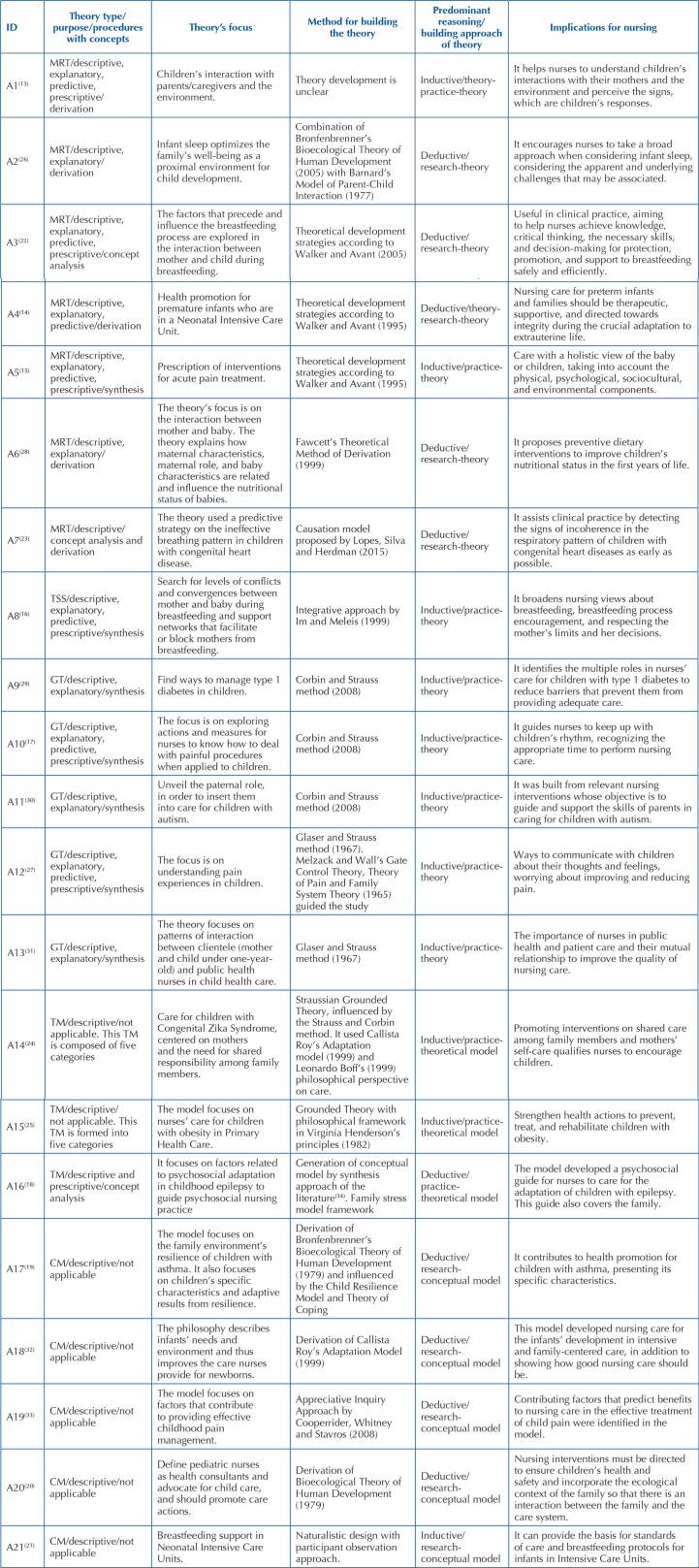
Categorization of nursing theories developed to meet children’s needs – João Pessoa, PB, Brazil, 2022, n = 21.

ID: identification; MRT: middle-range theory; TSS: Theory-Specific Situation; GT: Grounded Theory; TM: theoretical model; CM: conceptual model.

Chart 2 shows data organization related to nursing theories developed to meet children’s needs, which are found in the articles, according to the following variables: levels of theory development; purpose and uses; approaches to theory building; theory’s focus; method to build the theory; general procedures for building theories; theory development strategies; and nursing implications.

We identified seven middle-range theories or 33.33% of the total, five Grounded Theories (23.80%), three theoretical models (14.28%), three conceptual models (14.28%), and one theory-specific situation (4.76%).

We found nursing theories, in order to meet the following needs: infant feeding; childhood pain; a child with asthma; diabetes; obesity; epilepsy; Congenital Zika Syndrome; Autism Spectrum Disorder; theory for care in Intensive Care Units; health promotion in premature babies; procedures in Intensive Care Units; theory for nursing diagnosis ineffective breathing pattern in the child with congenital heart disease; sleep associated with child development; parent-child interaction; nurse-child relationship; and child consultation.

## DISCUSSION

Currently, there is a movement focused on developing theories/theoretical frameworks for nursing, aiming to reduce the gap between theory and professional practice by presenting more concrete concepts that can be applied and used in professional practice. The results pointed to the predominance of typology of middle-range nursing theories that are useful for this movement. Together with practical and situation-specific theories, they are closer to the empirical level^(1,34)^. This predominance of typologies more relative to the observed level also allows them to be directed to areas of activity, such as child health.

Identified in this research as “theoretical models” or simply as “models,” the theorized structures seem to corroborate the view that, in addition to theories, the models can also support the philosophy of nursing science, being especially important to present mechanisms, dynamics and other aspects of the modeled phenomena^(35)^. The structure of nursing knowledge includes elements such as metaparadigm, philosophy, conceptual models, grand theories, middle-range theories, and empirical indicators^(36)^; however, it appears that the empirical models are implicitly at an intermediate level between middle-range theories and empirical indicators. Having this information about the models is relevant, given that the so-called models of care can sometimes actually be theoretical constructions that more concretely represent conceptualizations related to the phenomena of care situations.

In child health, authors^(37)^ refer that nursing theories are still very recent, highlighting those of the context of hospitalization as an environment that requires more excellent professional performance through direct nursing care, as well as requiring a changing paradigm regarding the insertion of the family in the context of care.

In nursing, there is a tendency to use theories with a higher level of abstraction, as mentioned in the study on theoretical nursing conceptions in the care of the hospitalized child: scoping review^(37)^. Therefore, there is a need to develop less abstract theories focused on child health and disseminate and apply them in clinical practice, intending to promote nursing activities in the area.

Additionally, the review indicated that models with theorizing properties might not be seen with such property, which may indicate an underestimation of nursing theoretical development.

The needs prioritized in the findings referred to the risk of disease, cardiovascular disease, and the mother-child bond, with a general focus on breastfeeding and pain. Cardiovascular diseases are among the leading causes of hospitalization and death in Brazil and the world, demonstrating the essentiality of working both in the area of cardiovascular risk, preventing these conditions, and in the care of pre-established and diagnosed diseases from individuals’ childhood^(38)^.

Pain is a common aspect in most health priorities as a traumatic experience, requiring a unique look for its identification when working with children, through non-verbal signals, for instance, giving more excellent stability to children who do not have a clear understanding of the pain process^(39)^.

Another essential aspect of child care is the children’s relationship with the mother, father, family, and caregiver, as children do not have the autonomy to deal with their care and health. Nursing needs to offer more attention and aspects of care related to these people who are beyond the children. This process demonstrates the importance of the bonding relationship between children and their family and the uniqueness of this process, generally related to non-technological procedures and more directed to contact, dialogue and affection, as explained in the conceptions of these theories^(40)^.

The studies, as seen in the focus of the theories, emphasized the relational process where parents or caregivers were the primary sources of the report. However, in approaches focused on more specific phenomena, children’s responses were investigated, allowing the development of theories, for instance, in the condition of pain.

Middle-range theory, the most predominant type of theory in the research findings, has a lower level of abstraction than grand theories, which are also considered highly applicable in nursing practice. The middle-range theory has characteristics that differ from other theories, such as the delimitation of the number of concepts and exploration of a part of the phenomenon. They are based on philosophical perception, presenting their relationship with the real world, the theoretical part, and the methodological part of applying theory in professional practice, the practical part^(1)^.

Although the middle-range theory has practical applicability and can address phenomena limited to a particular human group, its use does not seem equally widespread among countries. Brazilian researchers have shown that its use is still restricted in Brazilian theses and dissertations compared to major theories^(10,41)^. It may relate to a recent diffusion of knowledge of middle-range theory in Brazil and the presumptive teaching at graduation that includes only nursing models and/or grand theories. Finally, a few textbooks translated into Brazilian Portuguese expose middle-range theory.

As for the method adopted for theory development, it is noteworthy that Grounded Theory, although not specific to nursing, was applied in knowledge production in nursing, producing primarily descriptive theories. However, the literature emphasizes that it is necessary to deepen and strengthen the justifications for choosing this method to conduct the studies, ensuring consensus, rigor, quality, and reliability of the studies produced by Grounded Theory^(42)^. Although the debate about the production properties of nursing theories by Grounded Theory may remain open among researchers who use the method, the potential of Grounded Theory is undeniable when a broader conception of what a nursing theory represents is employed.

The interpretation of this review’s findings points to a diversity of theory building methods, reiterating the metatheoretical statement that creativity is an essential process for theorists^(43)^.

Three main procedures with concepts were verified: analysis, derivation, and synthesis. The use of at least one of these procedures of concepts that was verified in the research reinforces the condition of the relevance of the concept in theorization. In theory building, conceptualization represents a relevant step that can either anticipate the elaboration of operational elements such as propositions and hypotheses, or be a product subsequent to data coding. Thus, the concepts are the primordial foundations in bulding the nursing theory’s conceptual structure. These three procedures have been confirmed in a significant number of researches of concepts that were and still are carried out^(4)^.

As for the predominant reasoning in theory development, there are induction and deduction strategies, and the second stands out, through which the researcher guides his data collection and develops insights by which it can change the questions to be asked to individuals, which will be focused on new theories to be created from pre-existing theories^(44)^. Along with abduction, such forms of reasoning seem to guarantee essential tools to build nursing theories, including what gives them an exciting position in training at the undergraduate level and, especially, at the *stricto sensu* graduate level.

The importance of developing and using nursing theories, and not exclusively care methodologies, can be defended. Theories can act as a reference or “lenses” that help in the care path design, defining essential aspects and health priorities that need standardization regarding the body of nursing knowledge. Thus, theories guide to care and offer an organized and sustained approach to knowledge^(45)^.

### Study Limitation

A limitation of this study was achieving a greater understanding of theories for children that were perhaps available until the 1990s. This is because few documents dated before 1992 were accessible, limiting the authors’ interpretations for the mentioned period.

However, the limitation of obtaining texts before the 1990s may have been influenced by the results profile, indicating the predominance of middle-range theory. Between the 1960s and 1980s, building major nursing theories that did not emphasize a particular population or human group predominated. Predominantly, from 1990 onwards, middle-range and specific situation theories with a lower level of abstraction, oriented to a particular phenomenon or population, began to be proposed, ultimately modifying theoretical nursing bases^(46)^.

### Implications for the Advancement of Scientific Knowledge for Health and Nursing

This research can contribute to developing new nursing theories in child health by presenting building methods, types of theory, purposes, procedures adopted to deal with concepts, predominant reasoning, and theory building approach. It can use various alternatives and metatheoretical, theoretical, and scientific knowledge to elaborate further or refine child-oriented theories available to theorists.

It has the potential to contribute to new nursing theory development in child health, by presenting building methods, types of theory, purposes, procedure adopted to deal with concepts, the prevailing reasoning and the theory building approach. It is understood that the presentation of these elements makes available to theorists a variety of alternatives and metatheoretical, theoretical and scientific knowledge that can be used for future elaborations or refinement of theories aimed at children.

## CONCLUSION

This review mapped the development of nursing theories to meet children’s needs. The identified theories showed a lower level of abstraction, with a predominance of the middle-range theory.

The research found theories about caring for children, their mothers, and their family members, contemplating the adaptation needs, breastfeeding of children, mother and child bond, cardiovascular system (congenital heart diseases), respiratory (asthma), digestive (nutritional needs, obesity), endocrine (diabetes), neurological (epilepsy, sleep), their health (autism) and pain management.

We identified a range of strategies for theorizing and manipulating concepts.

Finally, nursing theories and mapped models were outlined for the course of nursing care and aimed at meeting children’s needs.

## Financial support

 The present work was carried out with the support of the Coordination for the Improvement of Higher Education Personnel – Brazil (CAPES – *Coordenação de Aperfeiçoamento de Pessoal de Nível Superior*) – Financing Code 001. The research was also supported by the Brazilian National Council for Scientific and Technological Development (CNPq – *Conselho Nacional de Desenvolvimento Científico e Tecnológico*), under Process 305208/2020-9.
